# DAPEG, a Modular Peptidomimetic Concept for Intracellular Delivery: Architecture, Trajectory, and Outlook in the Context of Peptidomimetic Transfection Reagents

**DOI:** 10.1021/acsomega.6c06553

**Published:** 2026-10-14

**Authors:** Wiktoria Mallek, Gabriela Mirecka, Magdalena Wysocka

**Affiliations:** Faculty of Chemistry, 49646 University of Gdańsk, Wita Stwosza 63, Gdańsk 80-308, Poland

## Abstract

Cell-penetrating peptides (CPPs) remain a leading nonviral strategy for the intracellular delivery of polynucleotides and folded proteins, but native α-peptide CPPs are constrained by proteolysis, dose-dependent cytotoxicity, and endosomal entrapment. Peptidomimetic redesign addresses these limitations by replacing the α-amino-acid backbone, repositioning side chains, or embedding polyethylene glycol structurally. In this Topical Review, we present DAPEG, sequence-defined oligomers of 2,3-diaminopropionic acid bearing pendant polyethylene glycol arms developed at the University of Gdańsk, as a case study of how a single modular building block can migrate across functional categories within one decade, from fluorogenic protease substrates (2016, 2020) through plasmid DNA transfection with nonlipidated guanidinylated oligomers (2021) and delivery of active enzymes in breast-derived cell lines (2024) to lipidated plasmid DNA transfection in HEK-293T spheroid cultures (2025). To contextualize the chemistry, we survey eight peptidomimetic architecture classes now used as transfection reagents (peptoids and lipitoids, stearylated arginine-rich CPPs, PepFects, charge-altering releasable transporters, α-peptide/β-peptoid hybrids, PEGtides, modular peptide-PEG-oligocation constructs, peptidomimetic-functionalized lipids). We then examine DAPEG architecture and chronology; propose a mechanistic model for how N-terminal acyl-chain length tunes the balance between nucleic-acid condensation, particle size, and uptake (nonmonotonic and dependent on stoichiometry: the palmitoylated C16 variant gave the highest observed activity at a charge ratio of 1.5:1 and the acetylated C2 variant at 3:1 and 9:1, with C6 and C10 reaching the leading group only at 9:1); and acknowledge the six principal evidence gaps in the current DAPEG record as priority directions: the absence of in vivo data, unmapped cellular entry and endosomal escape, uncharacterized behavior in serum, unprofiled immunogenicity, a narrow cargo scope, and the absence of independent replication.

## Graphical abstract

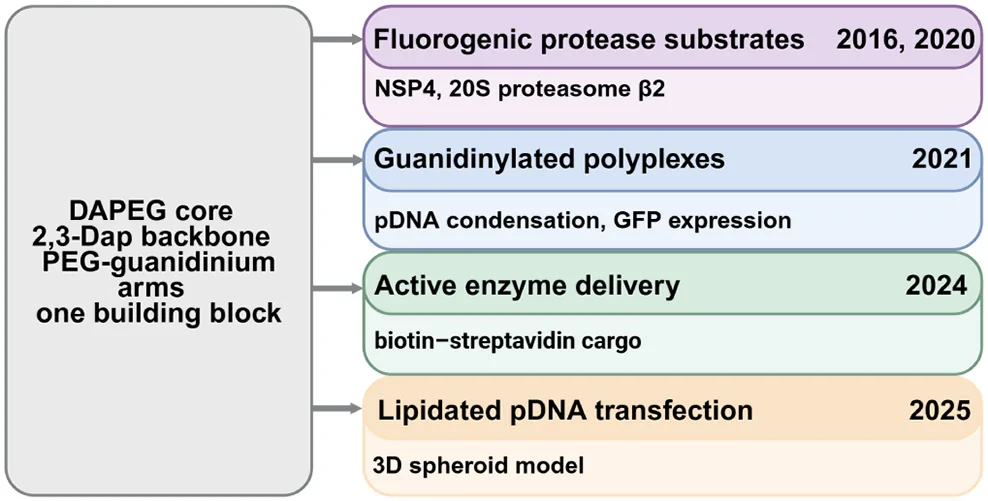

## 1 Introduction

Intracellular delivery of polynucleotides and folded proteins remains a central challenge in biomedicine. Among nonviral options, cell-penetrating peptides (CPPs) offer modular chemistry and low immunogenicity at modest doses. The field originated with the cellular uptake of HIV-1 Tat reported by Frankel and Pabo in 1988 ^1^ and the translocation of the *Drosophila* Antennapedia third helix described by Derossi and colleagues in 1994.^2^ The truncated Tat(48–60) basic domain reported by Vivès and colleagues in 1997 established that short cationic sequences are sufficient for membrane traversal.^3^ A foundational extension of this principle was the introduction of guanidinium-rich molecular transporters by Wender and colleagues in 2000, which demonstrated that the guanidinium headgroup, not the peptide backbone, is the determinant of cell uptake.^4^ A preclinical demonstration followed in 2012 with oligoarginine-taxol conjugates capable of overcoming drug resistance in patient-derived ovarian-carcinoma material.^5^ Recent applications span nasal,^6^ oncological,^7^ hepatic,^8^ and photochemically triggered delivery.^9^

Four limitations of native CPPs recur: proteolytic instability; dose-dependent cytotoxicity, often accompanied by immunogenicity; endosomal entrapment of cargo;^10,11^ and lack of intrinsic tissue selectivity. Peptidomimetic chemistry addresses each of these by replacing or reorganizing the building blocks of the peptide while preserving its functional logic.

This topical review presents the DAPEG concept, oligomers of 2,3-diaminopropionic acid (2,3-Dap) bearing pendant polyethylene glycol (PEG) arms, as a case study of how a single modular building block can migrate across functional categories within one decade. The primary literature calls these compounds polymers; we call them oligomers throughout because the chains are sequence-defined and monodisperse. We use the term *concept* rather than *platform* deliberately: the chemistry remains, at the time of writing, developed by a single laboratory and has not yet been independently replicated by other groups (see Section 3.4). To contextualize the chemistry, Section 2 surveys eight peptidomimetic architecture classes now used as transfection reagents. Section 3 then examines DAPEG architecture, chronology, mechanistic hypotheses, and current evidence gaps. Section 4 provides a comparative summary, including quantitative metrics, where available. Section 5 outlines the priorities and emerging frontiers.

### 1.1 Scope and Methodology of Literature Search

The primary corpus was retrieved from PubMed (queries executed in May 2026) using combinations of the descriptors “peptidomimetic”, “cell-penetrating peptide”, “transfection”, “siRNA delivery”, “gene delivery”, “peptoid”, “PEGtide”, “lipitoid”, and “2,3-diaminopropionic acid”. Inclusion criteria were peer-reviewed original papers and reviews reporting peptidomimetic vectors for nucleic-acid or protein cargo with in vitro or in vivo validation. The search returned approximately 60 records meeting the inclusion criteria across the period 1988–2026. Records primarily reporting building-block synthesis without delivery context (typical of *J. Org. Chem.*, *Org. Lett.*, *Tetrahedron)* were not systematically retrieved and may include chemistry not represented here; spot-check searches in Web of Science and SciFinder for selected building-block keywords did not return additional CPP-relevant records that were not already captured by the PubMed corpus, but a comprehensive synthesis-focused review is beyond the scope of the present manuscript. Where pre-2000 foundational papers were required for historical accuracy (notably Frankel and Pabo 1988, Derossi 1994, Vivès 1997, Boussif 1995, Wender 2000), they were retrieved by direct identifier lookup rather than by keyword search.

### 1.2 Rationale, Scope, and the Limits of What This Review Claims

A review devoted to a chemistry developed within a single laboratory requires explicit justification, and we state ours plainly. The case for examining DAPEG here does not rest on a claim of superior delivery performance; no such claim is supported by the present evidence, and Sections 3.4 and 5.1 set out in detail why. It rests instead on a structural observation that is verifiable from the published record independently of any efficacy comparison. A single orthogonally protected building block, Fmoc-L-Dap­(Mtt)-OH, has supported five distinct applications spanning three functional categories (molecular sensing, nucleic-acid delivery, and protein delivery) without alteration of the backbone chemistry or of the synthetic route. These are fluorogenic protease substrates (2016),^12^ library-scale proteasome substrates screened in the urine of bladder-cancer patients (2020),^13^ plasmid DNA transfection with nonlipidated guanidinylated oligomers (2021),^14^ streptavidin-scaffolded delivery of a catalytically active β-galactosidase tetramer (2024),^15^ and lipidated plasmid DNA transfection (2025).^16^ What migrates across these five applications is not a vector but a monomer: the two orthogonal nitrogen handles of 2,3-Dap allow the same solid-phase backbone to present a fluorophore-quencher pair, a combinatorial side-chain library, a biotin docking site, or a guanidinium-lipid combination. Modularity of this kind is a property of the building block and can be assessed from synthesis and characterization data alone, which is why it can be reviewed before efficacy data mature.

This argument is deliberately narrow, and we draw the boundary explicitly. Modularity is a necessary but not sufficient condition for a delivery platform. The nonviral delivery literature contains numerous chemistries that combined synthetic elegance with encouraging in vitro performance and have nonetheless not reached clinical use; attrition between in vitro promise and clinical translation is the rule in this field rather than the exception, and nothing in the DAPEG record exempts it from that base rate. We therefore position DAPEG throughout this review at the stage the evidence supports, a proof-of-concept chemistry with three delivery-related publications, no in vivo data, no mechanistic mapping of cellular entry or endosomal escape, no serum challenge data, no immunogenicity profile, and no independent replication (Section 5.1). We treat it as a case study in building block design rather than as a candidate vector. A reader seeking an assessment of DAPEG as a validated delivery platform will not find one here and should not; the record does not support it.

The timeliness of undertaking this now, rather than after in vivo validation, follows from the same logic. Sections 2 and 4 document that the peptidomimetic transfection field is not currently comparable across platforms: particle size, charge ratio, serum compatibility, and benchmark choice are reported inconsistently, and the “not reported in cited source” entries in Tables 1 and 2 (Section 4.2) mark the points at which cross-platform inference fails. An early-stage building block is precisely the object for which the design space remains open and for which the reporting standards proposed in Section 4.1 can still shape an experimental program prospectively rather than being applied retrospectively. Section 3.5 accordingly states the mechanistic model in a falsifiable form, Section 3.6 examines the serum question that any in vivo ambition must answer first, and Section 5 names the specific experiments that would confirm or refute the model. The value we claim for this review is that of a critical audit performed early enough to be actionable, not that of a verdict on a mature platform.

**Table 1:** Peptidomimetic Transfection Platforms: Comparative Summary

Class	Backbone	Cationic group	PEG role	Cargo	Particle size (nm)	Cytotoxicity profile	In vivo data	Ref.
Peptoid/lipitoid	Poly(*N*-substituted glycine)	Aminoethyl (peptoid); lysine analogs (lipitoid)	Phospholipid anchor (lipitoid)	pDNA, siRNA	50–100 (peptoid-pDNA);^18^ ∼120 (lipitoid-siRNA)^20^	Low; outperformed cationic lipids in some serum conditions^18^	Yes: rabbit vocal fold (siRNA)^21^	^18–22^
α/β-Peptide hybrid	α-peptide/β-peptoid alternation	Lys, homoarginine	Anionic lipid coat	siRNA	not reported in cited source	No detectable cytotoxicity or immunogenicity in vitro^34^	Not reported	^34^
PEGtide	Poly(ethylene oxide)	Guanidinium	Structural backbone	siRNA	not reported in cited source	No detectable toxicity in Calu-3, N41 ^35^	Not reported	^35^
Wagner peptide-PEG-Stp	α-peptide-PEG-Stp	Stp (oligoethanoamine)	Central spacer + shielding	pDNA	not reported in cited source	Not reported in cited source	Not reported in cited paper	^36^
Peptidomimetic-functionalized lipid	Lipid + peptidomimetic head	Variable	Optional LNP component	pDNA, siRNA, mRNA	Variable across the class; see refs	Variable across the class	Variable across the class	^37–39^
Stearylated R-rich CPP	α-peptide oligoarginine	Arg	N-terminal stearoyl	pDNA, siRNA, SCO	126 ± 2 (stearyl-R_8_/pDNA in DMEM, 2.5 μg/mL DNA);^24^ 1 400–5 100 (same peptide in HBS at 100 μg/mL DNA)^23^	Comparable to Lipofectamine and polyarginine;^23^ serum compatibility not established	Not reported in refs 23–26 (all in vitro)	^23–26^
PepFect (PF14 and analogs)	α-peptide (stearyl-TP10 derived)	Lys/Orn	N-terminal fatty acyl (C12–C22)	SCO, siRNA, pDNA, mRNA	∼70–150 (SCO nanocomplexes)^27^	Low in HeLa, mdx myotubes; serum-stable^27,30^	Yes: systemic gene delivery in mice;^28^ keratinocytes and in vivo mRNA^29^	^27–30^
Charge-altering releasable transporter (CART)	Oligo(α-amino ester)-*block*-lipid	Polycationic block (degrades to neutral diketopiperazines)	Lipid block (variable)	mRNA, pDNA, circRNA	∼100–200 (mRNA/CART)^31,32^	>99% transfection efficiency in HeLa with low toxicity^31^	Yes: multiple routes in mice (mRNA)^31–33^	^31–33^
**DAPEG**	**Sequence-defined poly(2,3-Dap) oligomer**	**Guanidinium (lipidated variants)**	**Orthogonal side-chain modification handle**	**Probes, enzymes, pDNA**	**140–600** **(compound alone);** **2 000–6 000** **(short chains + DNA);** **200–400** **(C12–C16 + DNA)** ^16^	No viability suppression to 50 μM (2D HEK-293T); to 10 μM in 3D spheroids (C2 variant to 5 μM);^16^ growth stimulation observed; see Section 3.3	**None reported to date**	^12–16^

**Table 2:** Head-to-Head Quantitative Comparison of Representative Peptidomimetic Vectors against Conventional Benchmarks (Lipofectamine 2000, PEI 25 kDa) and the Lipidated DAPEG Series^a^

System	Cell line	Cargo	Charge ratio, N:P or MR	Transfection/silencing	Cytotoxicity/viability	Size (nm)	ζ-potential (mV)	Serum-stable	Ref.
Lipofectamine 2000 (benchmark)	HeLa pLuc705	SCO (18-mer)	manufacturer protocol	Reference standard for SCO splice-correction assay^27^	Moderate to high; activity lost after solid formulation^27^	Not reported per-formulation	Not reported per-formulation	Reduced	^27^
Lipofectamine 2000 (benchmark)	HEK293	mRNA (eGFP, 2.5 μg)	2 μL reagent per 2.5 μg mRNA (optimum for this line)	∼91% eGFP+^49^	Viability not assayed; loss of efficiency at 6 μL in endothelial cells read as toxicity^49^	Not reported per-formulation	Not reported per-formulation	Reduced	^49^
PEI, branched (25 kDa benchmark; 800 kDa in ref 17)	3T3; HeLa and others^17^	pDNA	Broad optimum near 9 N:P; 6 to 20 within 1 order of magnitude^17^	Foundational reagent; activity persists across cell lines^17^	Low; no reduction in cell activity at the optimal transfection ratio^17^	Not reported in cited source per-formulation	Reported positive in cited source	Reduced	^17^
Stearyl-R_8_	COS-7 ^23^; NIH/3T3 ^24^	pDNA (luciferase)	2:1 (cation:anion optimum)	∼100-fold higher than unmodified R_8_ and comparable to LipofectAMINE in COS-7^23^; ∼ 3 orders of magnitude higher than unmodified R_8_ in NIH/3T3^24^	Comparable to Lipofectamine and polyarginine^23^	1 437 ± 1 273 (3 min) and 5 148 ± 3 377 (30 min) in HBS;^23^ 126 ± 2 in DMEM^24^	+19.3 ± 4.1 (stearyl-R_8_/DNA) vs +28.1 ± 1.1 (R_8_/DNA)^24^	Not tested; transfection performed in serum-free medium^23^	^23,24^
Stearyl-R_8_ (siRNA)	Primary rat neurons	siRNA	2:1 (stearyl-R_8_:siRNA optimum)	73 ± 2% Cy3-positive cells vs 83 ± 4% for Lipofectamine; EGFP silencing greater than Lipofectamine^25^	Metabolic activity 89–96% of control vs 73% for Lipofectamine^25^	Not numerically reported in cited source	Not reported	Not tested; transfection in supplement-free Neurobasal medium^25^	^25^
PepFect14 (C18 stearyl)	HeLa pLuc705	SCO	MR 5	Higher splice-correction than Lipofectamine 2000 ^27^	Low; serum-stable^27^	∼70–150 ^27^	Reported positive in cited source	Yes^27^	^27^
PepFect14 acyl-length SAR (C2 → C22)	HeLa pLuc705	SCO	MR 5	Activity emerges at C12, increases linearly to C22 ^30^	Low across the series^30^	No apparent difference C12–C22 ^30^	No apparent difference C12–C22 ^30^	Yes^30^	^30^
PEGtide (oligoArg-mimetic)	Calu-3, N41	siRNA	Not reported in cited source	Outperformed commercial controls and free R_8_ ^35^	No detectable toxicity^35^	Not reported in cited source	Not reported in cited source	Not reported	^35^
Peptoid 36-mer (Murphy 1998)	Multiple cell lines	pDNA	Not reported (charge-balanced)	Outperformed several cationic-lipid commercial reagents^18^	Low^18^	50–100 ^18^	Not reported in cited source	Yes^18^	^18^
Lipitoid-siRNA	Primary fibroblasts; HeLa	siRNA	Mass ratio (not N:P)	Effective knockdown,^19,20^	Low^20^	∼120 ^20^	Positive^20^	Partial	^19,20^
Zeng α/β-hybrid (palmitoylated)	HeLa	siRNA	Lipid-coated nanocomplex	Silencing comparable to commercial cationic lipids^34^	No detectable cytotoxicity or immunogenicity^34^	Not numerically reported in cited source	Net anionic after coating^34^	Yes^34^	^34^
CART D_13_:A_11_ (Wender 2017)	HeLa	mRNA	Charge ratio ∼ 10:1	>99% transfection in cultured cells^31^	Low; degrades to neutral diketopiperazines^31^	∼100–200 ^31^	Positive initially; decays to neutral^31^	Yes (used in vivo)^31^	^31^
CART (lipid-varied library)	Primary T-lymphocytes	mRNA	Charge ratio ∼ 10:1	Enhanced uptake into multiple lymphocyte types^32^	Low^32^	∼100–200 ^32^	Decays from cationic to neutral^32^	Yes^32^	^32^
Nonlipidated DAPEG parent, O2Oc-[Dap(GO2)]_*n*_-O2Oc-NH_2_, *n* = 4, 6, 8 (*n* = 6 most effective)	HB2, MDA-MB-231	pDNA (pmaxGFP)	1.5:1, 3:1	Fluorescence comparable to ViaFect at ∼ half its cytotoxicity; hexamer (*n* = 6) gave the highest observed fluorescence^14^	Tetramer and hexamer essentially nontoxic post-transfection; octamer ∼ 30% viability loss^14^	Flower-like complexes by AFM: ∼900 (hexamer), up to 2 350 (octamer)^14^	Not reported^14^	Not tested; transfection in serum- and antibiotic-free medium for 5 h^14^	^14^
**Lipidated DAPEG C2 (acetyl)**	**HEK-293T** **(2D + spheroids)**	**pDNA (pmaxGFP)**	1.5:1,3:1,9:1**tested**	**Highest at charge ratio**3:1**and**9:1**; second to C16 at**1.5:1^16^	**No viability suppression to 50 μM (2D); to 5 μM (3D spheroids)** ^16^	**∼2 000–6 000** **(short-chain group)** ^16^	**+15 to +20** **(complex);** **+18 to +35** **(free compound)** ^16^	**Not tested** ^16^	^16^
**Lipidated DAPEG C10 (decanoyl)**	**HEK-293T** **(2D + spheroids)**	**pDNA (pmaxGFP)**	1.5:1,3:1,9:1**tested**	**Not in the leading pair at**1.5:1**or**3:1**; second to C2 at**9:1^16^	**No viability suppression to 50 μM (2D); to 10 μM (3D spheroids)** ^16^	**2 000–6 000** **(short-chain group)** ^16^	**+15 to +20** **(complex);** **+18 to +35** **(free compound)** ^16^	**Not tested** ^16^	^16^
**Lipidated DAPEG C12 (lauroyl)**	**HEK-293T** **(2D + spheroids)**	**pDNA (pmaxGFP)**	1.5:1,3:1,9:1**tested**	**Below C2 at every ratio tested; not the leading compound at any ratio** ^16^	**No viability suppression to 50 μM (2D); to 10 μM (3D spheroids)** ^16^	**∼200–400** **(C12–C16 group)** ^16^	**+15 to +20** **(complex);** **+18 to +35** **(free compound)** ^16^	**Not tested** ^16^	^16^
**Lipidated DAPEG C16 (palmitoyl)**	**HEK-293T** **(2D + spheroids)**	**pDNA (pmaxGFP)**	1.5:1,3:1,9:1**tested**	**Highest at**1.5:1**; below C2 at**3:1**and**9:1^16^	**No viability suppression to 50 μM (2D); to 10 μM (3D spheroids)** ^16^	**∼200–400** **(C12–C16 group)** ^16^	**+15 to +20** **(complex);** **+18 to +35** **(free compound)** ^16^	**Not tested** ^16^	^16^

a Bolded rows are the lipidated DAPEG variants (the case study of this topical review). The four lipidated entries span both size groups and both ends of the acyl-chain series. Stoichiometries are given in the convention used by each primary source: DAPEG entries are charge ratios (Section 3.3), the PEI entry is a nitrogen-counted N:P ratio, and MR denotes a molar or mass ratio; these conventions are not interchangeable . Entries Marked *Not Reported in Cited Source* are discussed in Section 4.1.

## 2 Landscape of Peptidomimetic Transfection Reagents

Eight architecture classes are surveyed in this section and summarized, together with DAPEG, in Table 1; Figure 1 compares the backbone topologies of four representative classes alongside DAPEG. A ninth strategy, appending PEG to a preformed peptide CPP rather than embedding it as a building block, is briefly addressed in Section 2.9 as a comparator class, not as a ninth peptidomimetic.

**Figure 1:**
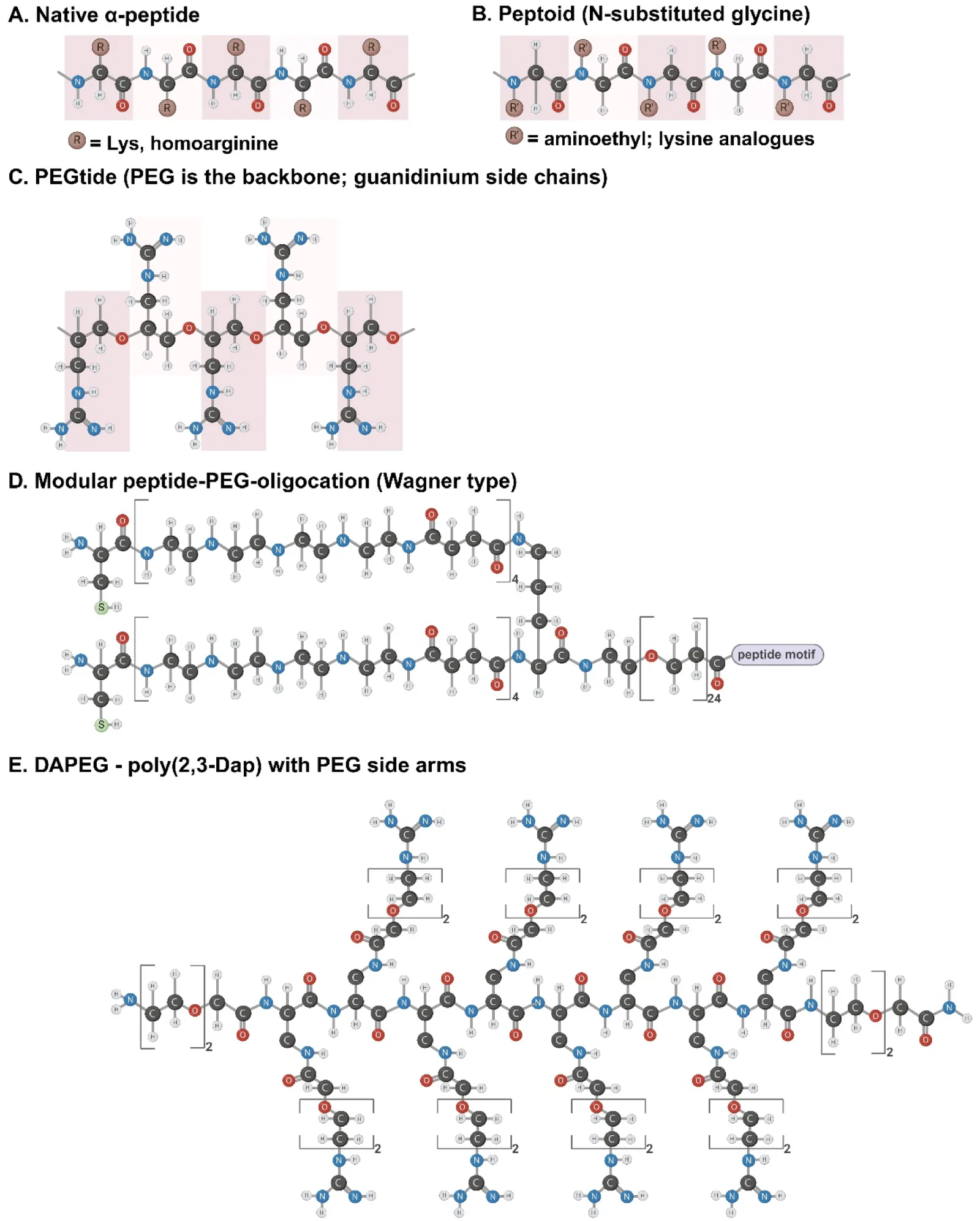
Topological comparison of peptidomimetic transfection vector backbones. (A) Native α-peptide with side chains projected from Cα. (B) Peptoid: side chains relocated to the backbone nitrogen, eliminating the backbone N–H and conferring proteolysis resistance. (C) PEGtide: poly­(ethylene glycol) backbone with pendant cationic (guanidinium) side chains. (D) Modular peptide-PEG-oligocation construct of the Wagner type, assembled by solid-phase synthesis. (E) DAPEG: poly­(2,3-Dap) backbone with polyethylene glycol arms appended to the side-chain nitrogen atoms, each arm terminating in a guanidinium headgroup. Structures are drawn as ball-and-stick repeating segments, not full-length molecules; atoms carry their element symbols and are colored by element (carbon, dark gray; nitrogen, blue; oxygen, red; hydrogen, pale gray; sulfur, green). Shading marks the repeating unit; square brackets with a subscript give the number of repeats, where n, m, and k are variables and the numerals 2, 4, and 24 give the repeat count in the published construct. R in (A) and R′ in (B) are the variable cationic side chains defined within the respective panels. Created in BioRender. Wysocka, M. (2026) https://BioRender.com/kryusi6.

### 2.1 Peptoids and Lipitoids

Peptoids are oligomers of N-substituted glycines in which side chains are appended to the backbone nitrogen rather than to Cα. The resulting tertiary-amide backbone resists proteolysis and accommodates submonomer combinatorial synthesis. Murphy and colleagues reported the first **peptoid-based** gene-delivery agents in 1998, within the polycationic delivery landscape already established by polylysine and the polyethylenimine (PEI) introduced by Boussif and colleagues in 1995,^17^ demonstrating that the N-substituted glycine backbone is competent for plasmid DNA condensation: a 36-mer with 12 aminoethyl side chains condensed plasmid DNA into 50–100 nm particles and outperformed several commercial cationic-lipid reagents, retaining activity in serum.^18^

Lipitoids, cationic peptoid head groups coupled to a phospholipid anchor, were introduced by Utku and colleagues for siRNA transfection in primary fibroblasts.^19^ Subsequent physicochemical and mechanistic characterization by Konca and colleagues implicated a lipid-raft- and dynamin-dependent uptake pathway, providing one of the better-characterized internalization profiles in the peptidomimetic literature.^20^ Mukudai and colleagues extended the approach in vivo with lipitoid-siRNA targeting Smad3 in a rabbit vocal-fold injury model, achieving statistically significant knockdown on day 1.^21^ A recent ring-opening polymerization strategy for tertiary-amine-pendant polypeptoids by Liao and colleagues with the Zuckermann group has produced pH- and thermoresponsive variants competent for siRNA transfection.^22^

### 2.2 Stearylated Arginine-Rich CPPs

N-terminal stearylation of cationic, arginine-rich CPPs constitutes one of the earliest and best-validated strategies for converting a membrane-translocating peptide into a transfection reagent. Futaki and colleagues established the strategy with stearyl-R_*n*_ oligomers (*n* = 4, 8, 12, 16) and showed that N-terminal stearylation increases transfection efficiency by approximately 2 orders of magnitude relative to the unmodified oligoarginine, reaching the same order of magnitude as Lipofectamine in COS-7 luciferase plasmid delivery; stearyl-R_8_ was the most effective member of this homologous series.^23^ A mechanistic dissection by Khalil and colleagues in NIH/3T3 cells showed that N-terminal stearylation shifts the internalization of the free peptide from a route largely insensitive to low temperature, hypertonic medium, and *N*-ethylmaleimide, characteristic of unmodified R_8_, to clathrin-mediated endocytosis and that atomic force microscopy shows the stearyl moiety condensing the plasmid completely into the complex core, whereas unmodified R_8_ leaves part of the DNA uncondensed; stearylation raised luciferase expression by about 3 orders of magnitude.^24^ Two details of that study bear directly on the DAPEG record. The pathway shift applies to the free peptide only: R_8_/DNA and stearyl-R_8_/DNA polyplexes were both blocked by low temperature and by hypertonic medium and therefore enter by the same clathrin-dependent route, so the transfection advantage of the stearylated peptide arises from condensation, surface adsorption, and nuclear delivery rather than from a change of entry portal.^24^ And the stearyl-R_8_/DNA complexes carried a lower zeta potential than unmodified R_8_/DNA complexes (+19.3 ± 4.1 mV against +28.1 ± 1.1 mV); the acyl chains partly shielded the cationic charge, in the same direction as that reported for the lipidated DAPEG series.^24^ Subsequent work extended the approach in two directions. In primary rat hippocampal neurons, stearyl-R_8_ reached 73% transfected cells against 83% for Lipofectamine, yet silenced EGFP more efficiently and preserved 89–96% of metabolic activity against 73% for Lipofectamine; uptake was endocytic, and the complexes colocalized with the lysosomal marker Lamp1.^25^ In octaarginine-modified nanoparticles, a high surface density of R_8_ shifts the dominant entry route from clathrin-mediated endocytosis to macropinocytosis and raises gene expression by about 3 orders of magnitude relative to low-density particles, without a corresponding increase in the amount internalized.^26^ The N-terminal acyl approach is the closest historical precedent for the lipidated DAPEG strategy of Mallek and colleagues (2025);^16^ the comparison developed in Section 2.3 is therefore essential rather than ornamental.

### 2.3 PepFects: N-Acyl SAR as the Closest Analog to Lipidated DAPEG

The PepFect family, developed by Langel and colleagues, is stearylated transportan derivatives engineered for noncovalent complexation with oligonucleotide and plasmid cargoes. PepFect14 (PF14; stearyl-AGYLLGKLLOOLAAAALOOLL-NH_2_, where O is ornithine) delivers splice-correcting oligonucleotides (SCOs) into HeLa pLuc705 cells at rates higher than Lipofectamine 2000 and, critically for translational utility, retains activity in serum and after solid-formulation drying.^27^ Charge- and fatty acid-modified PepFect analogs have achieved effective in vivo gene delivery with reduced toxicity.^28^ PF14-mediated mRNA delivery has subsequently been demonstrated in primary human keratinocytes and in vivo.^29^

Lehto and colleagues (2017) reported a systematic structure–activity study in which the N-terminal acyl chain of PF14 was varied from C2 to C22 and tested in the HeLa pLuc705 SCO splice-correction assay.^30^ Three findings of that study are directly relevant to the DAPEG record and require explicit comparison: (i) biological splice-correction activity emerged at C12 and increased linearly with each additional carbon up to C22; (ii) for the functionally active series (C12–C22), no apparent difference was observed in complex formation efficiency, hydrodynamic size, or zeta potential, that is, the structure–activity relationship was decoupled from particle-size and zeta-potential metrics across the active range; and (iii) the active range began at C12, whereas in the lipidated DAPEG series, C12 was not the highest performer at any charge ratio tested (Section 3.3); the boundary between the two DLS-defined DAPEG groups falls between C10 and C12, so the two systems are not aligned on a single structural transition.^16^

The contrast between the two SAR profiles is informative. In PF14, where the parent α-peptide is itself an amphipathic 21-mer with five cationic side chains (one Lys, four Orn) and a luciferase-restoration assay that reports cytoplasmic delivery of an 18-mer splice-correcting oligonucleotide, activity scales monotonically with acyl length above C12. In lipidated DAPEG, where the parent oligomer is a sequence-defined 2,3-Dap octamer carrying eight side-chain guanidinium groups and the assay reports cytoplasmic delivery of an ∼3.5 kb plasmid, activity is nonmonotonic and its ranking changes with the charge ratio: C16 gave the highest observed activity at 1.5:1 and C2 at the two higher ratios tested.^16^ The simplest unifying interpretation is that the cargo size sets the regime: for a small SCO, the dominant constraint is membrane interaction (favoring long acyl chains), whereas for a large plasmid, the dominant constraint becomes the balance between condensation (favoring longer chains) and dissociation in the endosomal compartment (favoring shorter chains), with an additional regime at the very-short end (C2) in which the acyl group contributes little and the chemistry behaves predominantly as a polycationic condenser. Direct head-to-head testing of the two systems on a common cargo set (SCO, siRNA, plasmid) would test this interpretation; we identify it as a priority in Section 5.1.

### 2.4 Charge-Altering Releasable Transporters (CARTs)

CARTs, developed by the Waymouth and Wender groups, are block oligomers prepared by organocatalytic ring-opening polymerization that consist of an initiator, one or more lipid blocks, and a polycationic oligo­(α-amino ester) block.^31^ Unlike all the systems reviewed in Sections 2.1–2.3, CARTs are not designed for permanent retention of their cationic character: the oligo­(α-amino ester) backbone undergoes a self-immolative intramolecular rearrangement to neutral diketopiperazine small molecules at physiological pH and temperature on a time scale of hours, releasing the polyanionic mRNA cargo. The original 2017 report demonstrated >99% transfection efficiency in cultured cells and robust protein expression in mice via multiple administration routes.^31^ A 2018 lipid-varied library extended the strategy to primary lymphocyte transfection, an application where lipid nanoparticles perform poorly,^32^ and a 2023 set of CARTs built on a β-amido carbonate backbone (bAC-CARTs) achieved enhanced mRNA transfection in primary T lymphocytes in vitro with highly selective spleen tropism in vivo.^33^

Two features of CARTs are conceptually relevant to the DAPEG records. First, the self-immolative release mechanism directly addresses one of the principal weaknesses of high-affinity cationic vectors, that tight cargo binding tends to be antagonistic to cytoplasmic release. The lipidated DAPEG record of Mallek and colleagues^16^ shows the same trade-off empirically (the C12–C16 variants condense DNA into compact particles, yet only C16, and only at the lowest charge ratio tested, reached the top of the transfection ranking, while C12 and C14 did so at no ratio); a programmed-release chemistry analogous to CART self-immolation, if installable on the Dap-PEG backbone, would be one productive direction for the next generation of DAPEG analogs. Second, the CART record demonstrates that a single polymer architecture, once mechanistically understood, can be migrated across cargoes (mRNA, plasmid DNA, circRNA) and across tissues (lymphocytes, tumors, spleen) by lipid-block variation, a translational trajectory absent from the current DAPEG record (see Section 3.4).

### 2.5 α-Peptide/β-Peptoid Hybrids

Zeng and colleagues developed palmitoylated α-peptide/β-peptoid peptidomimetics with systematic variation in lysine and homoarginine content.^34^ Anionic-lipid coating of the resulting siRNA nanocomplexes yielded net-anionic liposomes with silencing comparable to commercial cationic lipids and no detectable in vitro cytotoxicity or immunogenicity.

### 2.6 PEGtides

PEGtides, introduced by Hibbitts and colleagues, use poly­(ethylene glycol) itself as the main chain.^35^ Cationic side chains, guanidinium in the oligoarginine-mimetic variant, are installed by co- and postpolymerization functionalization of substituted ethylene oxides. PEGtide-siRNA outperformed commercial controls and free R_8_ in Calu-3 and mouse N41 cells without detectable toxicity. The innovation lies in embedding the PEG shielding element within the backbone rather than appending it postsynthetically.

### 2.7 Modular Peptide-PEG-Oligocation Constructs

Martin and colleagues in Wagner’s group assembled fully sequence-defined transfection vectors from three modules: a succinoyl-tetraethylene pentamine (Stp)-based oligocation DNA-binding domain flanked by cysteines, a central PEG24 spacer, and a peptidic targeting ligand (B6 for transferrin receptor or cyclic RGDfK for integrin αvβ3).^36^ The work established that Fmoc/Boc-protected oligoethanoamine acids can be used as ordinary building blocks in solid-phase synthesis, enabling modular, sequence-defined vectors with combined shielding, cargo binding, and receptor targeting.

### 2.8 Peptidomimetic-Functionalized Lipids

Cationic-lipid head groups have been replaced with peptidomimetic moieties to combine the formulation flexibility of lipid nanoparticles with the chemical tunability of peptidomimetics. Ehexige and colleagues developed a cationic peptidomimetically functionalized cholesterol with siRNA carrying capacity.^37^ Yu and colleagues showed that amine count on a lysine-based headgroup is nonmonotonically related to neural-cell transfection, with three amines optimal in their series.^38^ Sugimoto and colleagues designed a zwitterionic KK-(EK)-lipid that confers cell-penetrating behavior despite near-neutral net charge, illustrating that peptidomimetic headgroup chemistry can decouple cellular uptake from net cationic character.^39^

### 2.9 Comparator: Classical PEGylation and Conjugation of Preformed CPPs

Although not peptidomimetic in the strict sense, classical postsynthetic functionalization of preformed CPPs, which append PEG or other shielding and recognition elements to an α-peptide CPP rather than embedding them as building blocks, provides a meaningful comparator class. Three examples illustrate the strategy. Osman and colleagues reported PEGylated GET peptides for pulmonary gene therapy.^40^ Bhatt and colleagues described PEGylated dendrimer-CPP conjugates for paclitaxel anticancer delivery, with α-tocopherol functionalization for enhanced cellular uptake.^41^ At the periphery of this category, peptide-PEG conjugates with antimicrobial-peptide payloads have been used for surface-immobilized biosensing applications, exemplified by the DAL-PEG-DK5-PEG-OH conjugate for electrochemical lipopolysaccharide detection on gold electrodes reported by Kosikowska-Adamus and colleagues.^42^ That work uses peptide-PEG chemistry adjacent to, but not strictly identical with, DAPEG, and is positioned here as a comparator of the broader peptide-PEG conjugate space rather than as a DAPEG application.

These approaches differ from the peptidomimetic strategies in Sections 2.1–2.8 in that the PEG element is added postsynthetically to a peptide whose backbone remains canonical, rather than being structurally embedded.

## 3 DAPEG: Case Study

### 3.1 Architecture and Design Logic

The DAPEG building block was introduced in 2016 by Wysocka and colleagues:^12^ peptidomimetics composed of repeating 2,3-diaminopropionic acid (2,3-Dap) residues with side-chain nitrogen atoms modified by heterobifunctional polyethylene glycol chains. The acronym denotes the “diaminopropionic acid-PEG” architecture.

The chemical architecture of the DAPEG building block is shown in Figure 2. Three structural features distinguish DAPEG from the other classes reviewed in Section 2:

the main chain is peptide-like (Dap-derived), preserving compatibility with Fmoc solid-phase peptide synthesis on standard resins (see Scheme 1);
the side chain is an entire PEG segment, structurally embedded in every monomer of the backbone rather than appended postsynthetically;
each monomer carries two orthogonal nitrogen handles (α-amino and side-chain β-amino), enabling unusually dense and orthogonal modification at every residue of the oligomer.

**Scheme 1:**
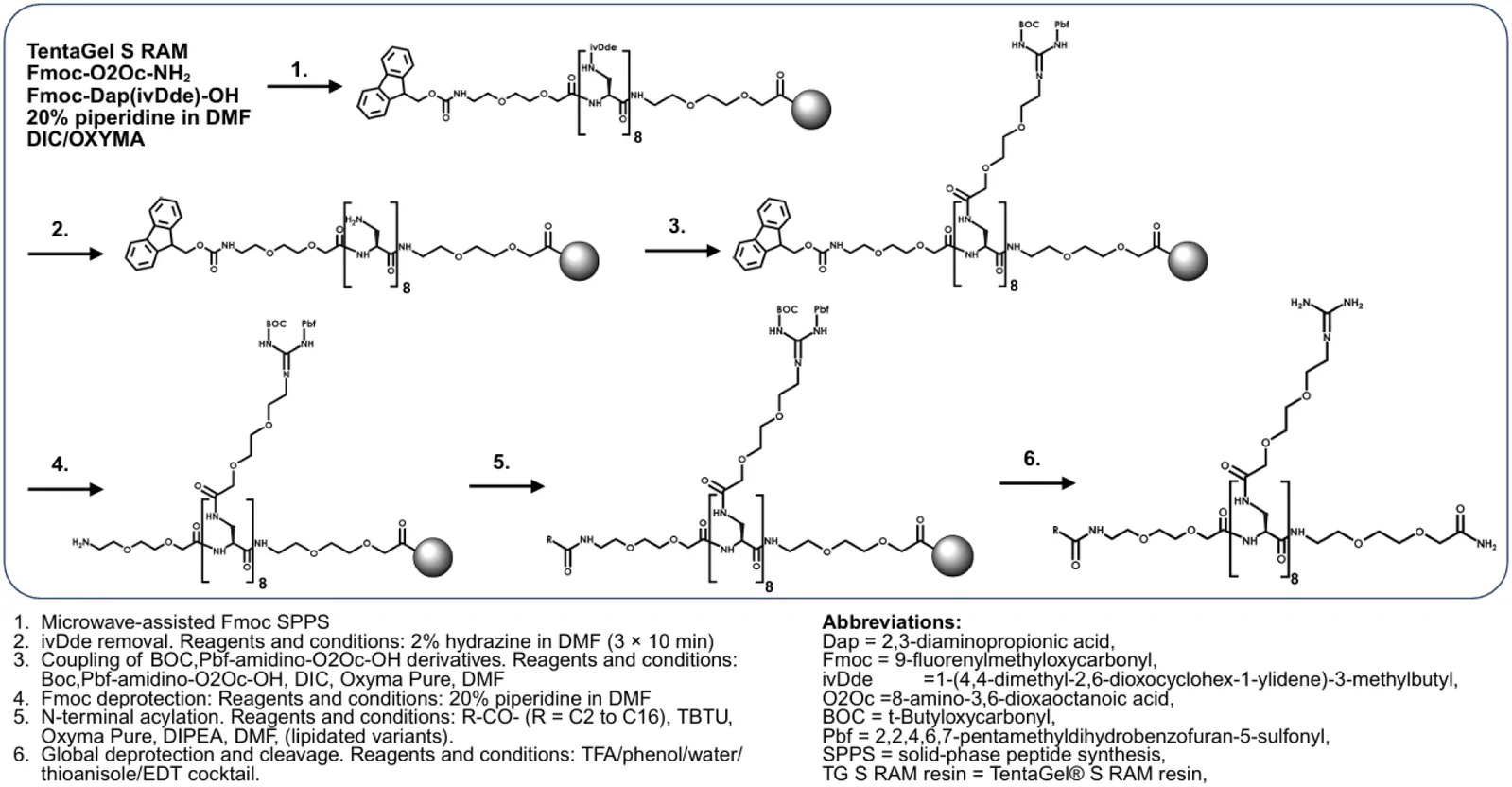
Synthesis of DAPEG Analogs^a^

a The route drawn is the procedure used for the lipidated series; ref^16^ describes the synthesis of those compounds by reference to ref^14^ and does not itself restate the protecting-group chemistry or the coupling reagents. Step 1: microwave-assisted Fmoc SPPS on TentaGel S RAM resin, starting from Fmoc-O2Oc–OH and the orthogonally protected 2,3-Dap building block Fmoc-L-Dap­(ivDde)-OH, with DIC/Oxyma Pure activation and 20% piperidine in DMF for Fmoc removal in each cycle; the ivDde group on the β-amino nitrogen is stable to piperidine and thus survives backbone assembly. Step 2: ivDde removal with 2% hydrazine in DMF (3 × 10 min), exposing the eight β-amino nitrogens at once. Step 3: coupling of the Boc/Pbf-protected PEG-guanidinium arms (drawn as Boc,Pbf-amidino-O2Oc-OH) with DIC/Oxyma Pure in DMF. Step 4: removal of the N-terminal Fmoc group with 20% piperidine in DMF. Step 5: for lipidated variants, N-terminal acylation with the corresponding fatty acid, C2 acetic to C16 palmitic, using TBTU/Oxyma Pure/DIPEA in DMF; of this step, ref^16^ records only that a twofold excess of the acid was used. Step 6: global deprotection and cleavage from the resin in TFA/phenol/H_2_O/thioanisole/EDT (82.5:5:5:5:2.5, v/v), followed by lyophilization, desalting on a C8 SPE cartridge, and confirmation of identity and purity by RP-UPLC and high-resolution MALDI MS (2,5-dihydroxybenzoic acid matrix). The earlier DAPEG work follows the same strategy with a different β-amino protecting group and correspondingly different conditions: refs 12 and 14 use Fmoc-L-Dap­(Mtt)-OH, with the acid-labile 4-methyltrityl (Mtt) group removed by dilute trifluoroacetic acid in dichloromethane in the presence of a scavenger (2% TFA with 1% triisopropylsilane in^12^; 1% TFA with 2% 1,2-ethanedithiol in^14^), backbone assembly performed manually by the portioning-mixing method in^12^, and the arms coupled with DIPCI/HOBt in^12^ and TBTU/Oxyma/DIPEA in^14^; cleavage in^12^ uses TFA/phenol/triisopropylsilane/H_2_O (88:5:2:5, v/v) and the crude products are analyzed by RP-HPLC and MALDI MS with an α-cyano-4-hydroxycinnamic acid matrix. Created in BioRender. Wysocka, M. (2026) https://BioRender.com/oa0nywq.

**Figure 2:**
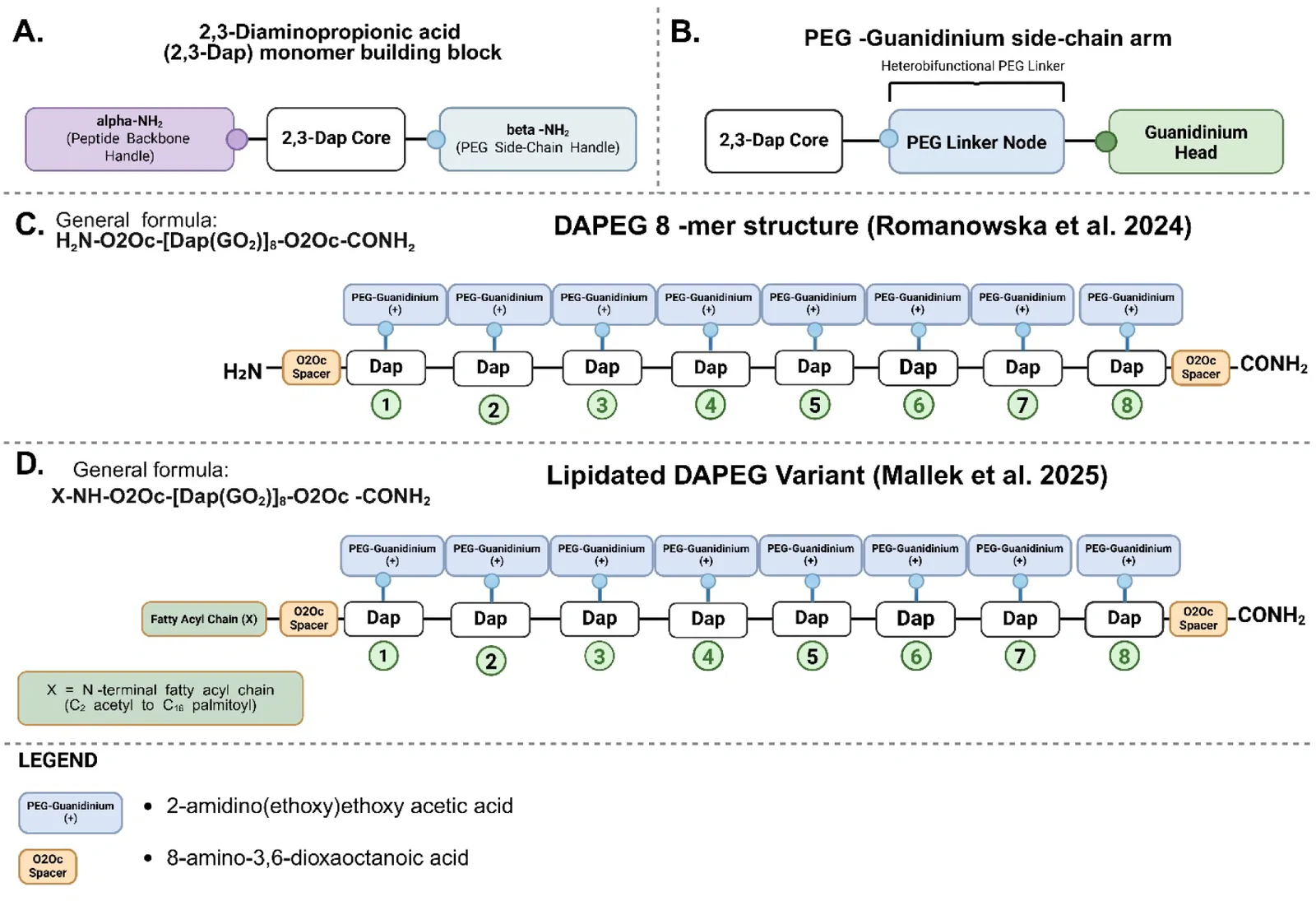
Architecture of the DAPEG building block, drawn as a connectivity schematic rather than as a full structural formula. (A) 2,3-Diaminopropionic acid (2,3-Dap) monomer showing the two orthogonal nitrogen handles: α-NH_2_ for peptide-bond formation and β-NH_2_ for PEG-arm attachment. (B) Heterobifunctional PEG side-chain arm terminating in a guanidinium headgroup, attached to the β-amino nitrogen of Dap; the arm carries two ethylene glycol units in the published variants. (C) DAPEG 8-mer of general formula H_2_N-O2Oc-[Dap­(GO2)]_8_-O2Oc-CONH_2_ (Romanowska et al. 2024),^15^ with eight PEG-guanidinium arms on the Dap backbone and a hydrophilic O2Oc spacer (8-amino-3,6-dioxaoctanoic acid) at each terminus; the same nonlipidated scaffold is the parent compound of the 2021 transfection series^14^ and of the lipidated variants shown in (D). (D) Lipidated DAPEG variant (Mallek et al. 2025):^16^ X-NH-O2Oc-[Dap­(GO2)]_8_-O2Oc-CONH_2_, where X is an N-terminal fatty acyl chain ranging from C2 (acetyl) to C16. Created in BioRender. Wysocka, M. (2026) https://BioRender.com/zlbmr7v.

The PEG element in DAPEG is not a classical PEGylation shield against serum nucleases; its role is to provide an orthogonal modification handle that can carry guanidinium groups, biotin tags, fluorophores, or further chemistry depending on the application. Three terms are kept distinct throughout this review. PEG-containing describes any architecture incorporating ethylene glycol units, including DAPEG; PEGylated describes the covalent attachment of a PEG chain to a therapeutic entity in the pharmacological sense; and PEG-shielded describes a dense surface corona of sufficient molecular weight to suppress protein adsorption. DAPEG is PEG-containing but is neither PEGylated nor PEG-shielded in this sense. This is a different functional role from the PEG used in clinical PEGylated drugs or in PEG-shielded LNPs, and it should not be read as conferring serum shielding; the consequences of the distinction are examined in Section 3.6.[Sec sec3.6].

The synthesis of DAPEG analogs is built on Fmoc SPPS and turns on one design choice: the 2,3-Dap building block carries two orthogonally removable protecting groups, one on each nitrogen (Scheme 1). An Fmoc group on the α-amino nitrogen is cleaved with piperidine in every SPPS cycle, while the β-amino nitrogen carries a group that survives those cycles and is removed only once the backbone is complete, exposing all eight β-amino nitrogens at once for attachment of the PEG-guanidinium arms. For lipidated variants, the N-terminal α-amino group is acylated with the corresponding fatty acid (C2 to C16) after the final Fmoc removal and before cleavage. The strategy preserves orthogonality and allows the same SPPS backbone to be diversified at three positions (α-NH_2_, β-NH_2_, and C-terminus). The choice of β-amino protecting group, and with it the reagents and conditions, has changed over the course of the work: the route used for the lipidated series and drawn in Scheme 1 employs the hydrazine-labile ivDde group, whereas the published procedures of refs^12^ and^14^ use the acid-labile Mtt group. Reference^16^ describes the synthesis of the lipidated compounds by reference to ref^14^ and does not restate the protecting-group chemistry. Both routes are set out in the caption to Scheme 1 and the protocols in full in refs 12,14 , and^16^.

### 3.2 Sensing Applications (2016, 2020)

The first DAPEG library^12^ yielded fluorogenic substrates for human neutrophil serine protease 4 (NSP4) with high sensitivity and selectivity; Figure 3 places this and the later applications in chronological order. An internally quenched library of general formula ABZ-X_4_-X_3_-X_2_-Arg-ANB-NH_2_, carrying a 2-aminobenzoic acid (ABZ) donor and an amide of 5-amino-2-nitrobenzoic acid (ANB-NH_2_) quencher and built from 18 building blocks at each of the three variable positions with the P1 arginine held fixed, gave approximately 6 000 compounds. Iterative deconvolution against NSP4 identified ABZ-Dap­(O2­(Cbz))-Dap­(Cbz)-Dap­(GO1)-Arg-ANB-NH_2_ as the sequence most susceptible to NSP4-mediated proteolysis, with *k*_cat_/K_M_ = 1.3 × 10^5^ M^–1^ s^–1^, 10-fold above the substrate reported by Lin and colleagues and 4-fold above the most potent NSP4 substrate then available. The compound was hydrolyzed only at the Arg-ANB bond, was not processed by cathepsin G, neutrophil elastase or proteinase 3 at 10^–8^ M, and gave a detectable signal at 10^–11^ M enzyme. In silico analysis suggested the peptidomimetic substrate engaged enzyme subsites that may be difficult to address with conventional α-peptide probes: docking placed the Dap­(GO1) guanidinium group in the S4 pocket formed by His99, Trp172, His175, and Phe215, a site not reached by the shorter proteinogenic side chains of earlier substrates. A 2020 follow-up^13^ applied the same chemistry to the trypsin-like β2 subunit of the human 20S proteasome, identifying, from 18 sublibraries totaling approximately 6 000 compounds, the substrate ABZ-Dap­(O2­(Cbz))-Dap­(GO1)-Dap­(O2­(Cbz))-Arg-ANB-NH_2_ with *k*_cat_/K_M_ = 7.61 × 10^7^ M^–1^ s^–1^, more than 2 orders of magnitude above the most potent β2 substrate reported previously, a detection limit near 10^–11^ M and, unusually, no requirement for SDS as an artificial activator; the substrate discriminated urine from bladder-cancer patients (*n* = 8) from healthy urine (n = 5). The library scale establishes that the chemistry is compatible with combinatorial workflows.

**Figure 3:**
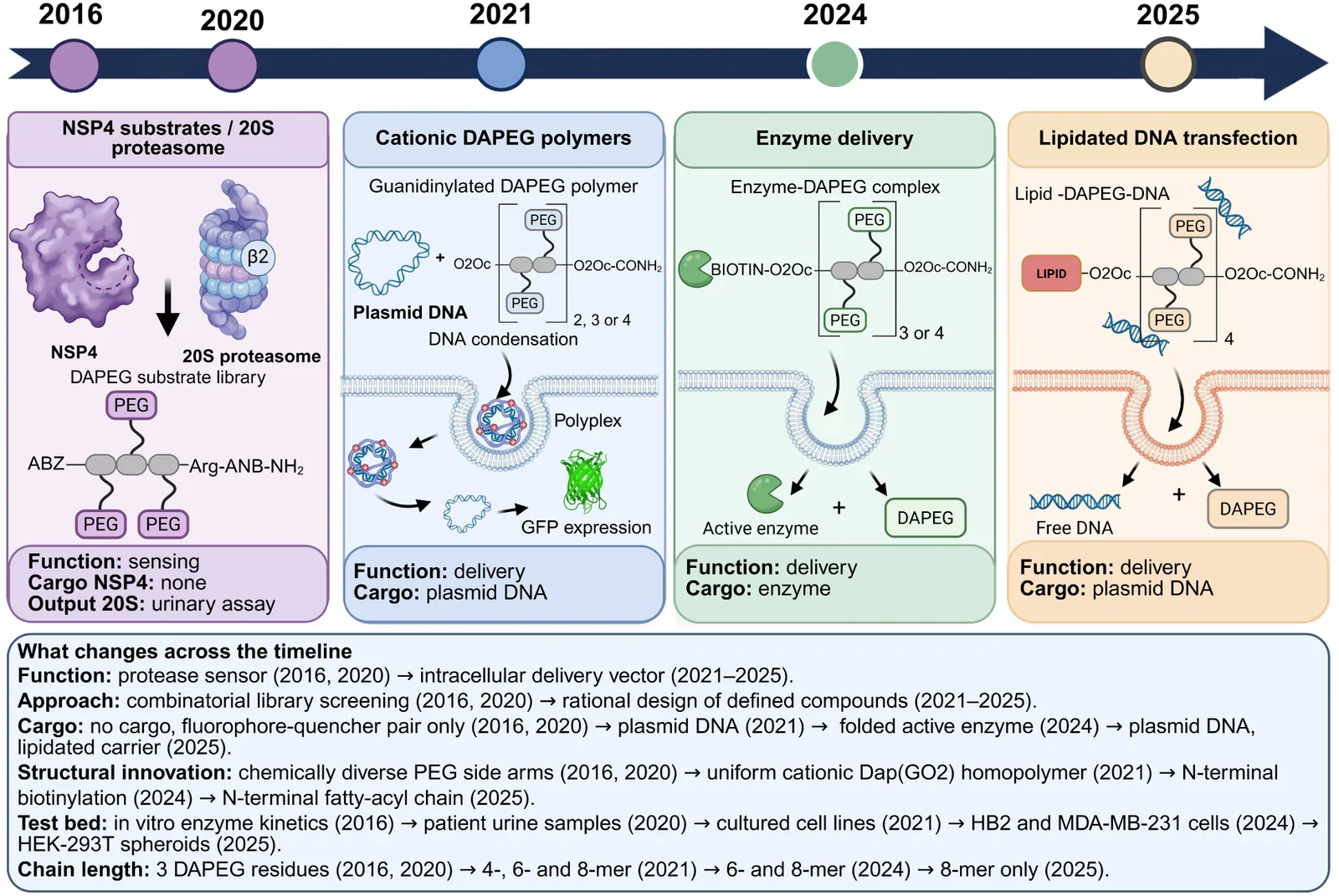
Chronological evolution of the DAPEG chemistry from 2016 to 2025. The same 2,3-Dap building block has been applied successively to fluorogenic protease substrates (NSP4, 2016; 20S proteasome β2 subunit, 2020), to plasmid DNA transfection with nonlipidated guanidinylated oligomers of chain length *n* = 4, 6, and 8 (2021), to delivery of a catalytically active enzyme docked through an N-terminal biotin to a streptavidin-β-galactosidase fusion (2024), and to plasmid DNA transfection with N-terminally lipidated 8-mers (2025). Created in BioRender. Wysocka, M. (2026) https://BioRender.com/6u6sf99.

### 3.3 Transition to Intracellular Delivery (2021, 2024, 2025)

The move from sensing to delivery began in 2021. Romanowska and colleagues^14^ showed that nonlipidated guanidinylated DAPEG oligomers of general formula O2Oc-[Dap­(GO2)]_*n*_-O2Oc-NH_2_, with *n* = 4, 6, and 8, condense plasmid DNA and act as transfection agents. The oligomers bound double-stranded DNA with affinities in the low-micromolar range (0.3, 3.4, and 0.19 μM for *n* = 4, 6, and 8, respectively) and were not cytotoxic to HB2 and MDA-MB-231 cells at the concentrations used.

Composition in this Review is expressed as the charge ratio, that is, the number of positive charges contributed by the peptidomimetic per phosphate charge on the nucleic acid. Both primary DAPEG delivery studies varied this quantity, although each labels it N/P. Reference^14^ gives both readings in a single sentence, defining the ratio as charge of the peptidomimetic per charge of the plasmid and then glossing N:P as nitrogen atoms per phosphate group, while reporting its own values elsewhere in the paper as charge ratios; ref^16^ defines it only as charge of the peptidomimetic per charge of pmaxGFP.^14,16^ The two conventions are not interchangeable for guanidinium carriers because a guanidinium group contributes three nitrogen atoms but only one positive charge, so a ratio obtained by counting nitrogen atoms would be up to threefold higher for the same composition. Ratios quoted for polyamine carriers such as PEI in Tables 1 and 2 follow the convention of their own sources, in which N:P counts nitrogen atoms irrespective of protonation, and are therefore not directly comparable with the DAPEG charge ratios.

Selected compounds transfected a GFP-encoding plasmid (pmaxGFP, 3 486 bp) at charge ratios of 1.5:1 and 3:1, benchmarked against the commercial reagent ViaFect: the hexamer matched the commercial control in fluorescence intensity, while the control killed about half the cells; the tetramer and the hexamer were essentially nontoxic after transfection; and the octamer reduced viability by about 30%. Atomic force microscopy showed that chain length governs the organization of the resulting flower-like DNA nanostructures: the hexamer formed assemblies averaging approximately 900 nm, and the octamer bridged several plasmids into connected coils reaching 2.35 μm. The most effective hexamer is, notably, the weakest binder of the three, and the authors attribute the pattern to complex size rather than to affinity: the tetramer condenses a plasmid intramolecularly into small particles but penetrates cells poorly on its own; the octamer forms assemblies too large for uptake; and only the hexamer lands in a size range compatible with membrane crossing. This is the first appearance in the DAPEG record of the size-affinity trade-off developed for CARTs in Section 2.4 and for the lipidated series in Section 3.5: tighter cargo binding does not translate monotonically into higher transfection.

The same study also reports the only inhibitor-based uptake data in the DAPEG record: internalization of the fluorescent hexamers was abolished at 4 °C and reduced by chlorpromazine and cytochalasin D but not by methyl-β-cyclodextrin, indicating a clathrin- and actin-dependent endocytic route for the free oligomer. This study established the guanidinylated Dap­(GO2) homo-oligomer as a functional nucleic-acid delivery vector, the nonlipidated parent of the lipidated series described below, and identified 2021, not 2024, as the point at which the DAPEG chemistry first crossed from sensing into intracellular delivery.

Building on this delivery precedent, Romanowska and colleagues^15^ next reported the first application of guanidinylated, biotinylated DAPEG oligomers to intracellular delivery of active enzymes. Two compounds, a 6-mer (Compound 1) and an 8-mer (Compound 2) of biotinylated DAPEG, were tested across four breast-derived cell lines: HB2 (nontumorigenic), MDA-MB-231 (triple-negative), SK-BR-3 (HER2+), and T47D (luminal A). No significant viability reduction was observed up to 20 μM in HB2 and MDA-MB-231, but SK-BR-3 and T47D showed significant viability reduction already at 10 μM and were therefore excluded from the β-galactosidase delivery experiments. Complexes with streptavidin-FITC and a streptavidin-β-galactosidase fusion crossed the membranes of both HB2 and MDA-MB-231 cells; intracellular β-galactosidase activity was 2–6-fold elevated in cells treated with biotinylated complexes versus nonbiotinylated controls, demonstrating that the cargo arrived in a functional state. The biotin moiety, coupled to the N-terminal amino group of the oligomer with Bt-NHS/DIPEA, served as the noncovalent docking site for the streptavidin-enzyme cargo (high-affinity biotin–streptavidin interaction, K_d_ in the subpicomolar range), allowing the vector to carry an intact streptavidin tetramer of approximately 60 kDa fused to β-galactosidase, itself a homotetramer of 1023-residue subunits. The 8-mer (Compound 2) was a more efficient transporter than the 6-mer (Compound 1) at both of the stoichiometric ratios tested, 1:2 and 1:4. Vesicular and membrane intracellular localization was confirmed by immunostaining; quantitative endosomal-escape data remain to be generated.

Mallek and colleagues^16^ extended the chemistry to plasmid DNA transfection. Eight lipidated variants of an octameric L-2,3-Dap parent compound bearing pendant guanidine groups, differing in acyl-chain length from C2 (acetyl) to C16 (palmitoyl), were synthesized and tested in HEK-293T cells cultured both as 2D monolayers and as 3D spheroids, the latter taken by the authors to reflect the native cellular environment more closely. Viability was not suppressed relative to untreated controls up to 10 μM in spheroid culture (up to 5 μM for the C2 acetyl variant) or up to 50 μM in the 2D assay; the caveat attached to that readout is set out at the end of this section.

Direct biophysical characterization by dynamic light scattering (DLS) revealed two distinct mechanistic regimes governed by acyl chain length: the parent compound and every variant up to and including C10 formed large aggregates of 2 000–6 000 nm with plasmid DNA, while the C12, C14, and C16 variants gave compact particles of 200–400 nm. This division is stated compound by compound in the results of the primary study and can be directly read from its scattering data. The scattering series was recorded at a single charge ratio of 1.5:1. Compounds alone (without DNA) had particle sizes of 140–600 nm. Zeta potential measurements confirmed the electrostatic nature of the compound-DNA interaction: free compounds carried +18 to +35 mV surface charge, reduced to +15 to +20 mV in the DNA complexes, a partial neutralization consistent with electrostatic complexation that nevertheless leaves the complexes net cationic.

Transfection efficiency was nonmonotonic in chain length, and the ranking changed with the cation-phosphate stoichiometry rather than being fixed by chain length. At the lowest charge ratio tested, 1.5:1, the highest fluorescence was obtained with the palmitoylated C16 variant, followed by the acetylated C2 variant. At a charge ratio of 3:1, the C2 variant gave the highest signal, and no other compound reached half of it. At a charge ratio of 9:1, the C2 variant was again the highest, followed by the C6 and C10 variants. The parent compound, which carries free N-terminal amino groups, was the least efficient at every ratio, which the authors read as evidence that masking the positive charge at that position is important. No statistical comparison between chain lengths was reported; therefore, these are the highest observed values rather than established optima. The nonmonotonic relationship is consistent with the proposition that acylation modulates a balance among DNA condensation, particle size, and membrane traversal kinetics, rather than simply enhancing membrane integration.

One further observation in that study bears on any future in vivo use and is not a delivery result at all: rather than a conventional dose–response decline, the compounds stimulated growth, with spheroid cell numbers exceeding untreated controls several times over for the longer-chain variants and a biphasic profile for the shorter ones. The authors note that uncontrolled stimulation of cell growth is itself a risk factor, and the effect was unexplained. Whatever its cause, it means that viability readouts in this system cannot be interpreted as a simple absence of toxicity.

### 3.4 Distinctive Features and Open Questions

Four properties distinguish DAPEG: a single nonproteinogenic building block (2,3-Dap) with two orthogonal nitrogen handles; structural embedding of PEG as a side chain; demonstrated library-scale synthesis on the order of 6 000 members; and an unusual cross-functional trajectory from sensing to delivery within one decade.

Six evidence gaps remain in the current DAPEG record and are acknowledged here without minimization. (i) All published delivery experiments are in vitro; no in vivo data on biodistribution, efficacy, or safety have been reported. (ii) Cellular entry is only partly mapped: an inhibitor panel has been applied to the free nonlipidated hexamers, indicating clathrin- and actin-dependent endocytosis,^14^ but no such panel has been applied to any DAPEG-cargo complex or to the lipidated series, and the octaarginine record shows that a free peptide and its polyplex can use different entry routes.^24^ (iii) Endosomal escape efficiency has not been quantified. (iv) Innate and adaptive immunogenicities remain unstudied. (v) The chemistry has been developed by a single laboratory; independent replication is an important next step. (vi) Behavior in serum has not been characterized (Section 3.6). Each gap is expanded in Section 5.1. They remain especially pertinent for DAPEG, given its short delivery-related record of three papers between 2021 and 2025.

### 3.5 Proposed Mechanistic Model and Testable Hypotheses

The nonmonotonic dependence of plasmid DNA transfection efficiency on acyl-chain length reported by Mallek and colleagues,^16^ in which the ranking of the variants changes with the cation-phosphate stoichiometry rather than following chain length, invites mechanistic interpretation. Here, we outline a working model and propose the experiments needed to confirm or falsify it.

#### 3.5.1 Working Model

The lipidated DAPEG vector functions in three sequential roles: (i) electrostatic condensation of the nucleic-acid cargo through the eight guanidinium groups on PEG side arms; (ii) modulation of particle size and surface hydrophobicity through the N-terminal acyl chain; (iii) membrane interaction and intracellular release. The orthogonal nitrogen handles of 2,3-Dap allow these roles to be assigned to chemically distinct positions on the same monomer, guanidinium-PEG arms on β-NH_2_ and lipid on the α-NH_2_ terminus, without interference.

#### 3.5.2 Two-Group Hypothesis (Supported by DLS)

Direct DLS measurements by Mallek and colleagues^16^ resolved the lipidated DAPEG series into two mechanistic groups. **Group I** comprises short-chain variants, up to C10, that on mixing with plasmid DNA give aggregates of 2 000–6 000 nm, consistent with bridging of multiple plasmid molecules by short, weakly hydrophobic vectors that cannot collapse the complex efficiently. **Group II** comprises long-chain variants (C12–C16) that form compact 200–400 nm condensates. One hypothesis consistent with this observation is cooperative folding of a single plasmid by a single vector molecule, driven by the hydrophobic packing energy of the longer acyl chains; the DLS data alone do not establish either the stoichiometry or the folding mechanism. On the basis of the compound-by-compound data reported in the results of the primary study, the transition falls between C10 and C12. Because the DLS series was recorded at a single charge ratio of 1.5:1, the reproducibility of the transition across the charge ratios is untested.

The size data cannot, however, be mapped onto the transfection ranking, and it is worth being explicit about this. Particle sizes were measured at a charge ratio of 1.5:1. At that ratio, the best-performing compound is C16, which forms compact particles, while the second best is C2, which forms micrometer-scale aggregates; the two mechanistic groups are therefore represented at the top of the ranking by one compound each. At charge ratios of 3:1 and 9:1, where C2 and then C2 followed by C6 and C10 dominate, no size data exist. Neither group is therefore superior as a class on the evidence available, and the complex size at one stoichiometry does not predict activity at another. What the record shows is that the relation between cargo compaction and delivery is not monotonic. The same is true in the nonlipidated series, where the hexamer was simultaneously the weakest DNA binder and the most effective transfection agent. A defensible reading is that particle size is permissive rather than optimizable within this chemistry and that the rate-limiting step lies after complex formation; testing that reading requires size measurements at each of the three charge ratios, which is the first experiment we would recommend.

One further observation in the same study bears on reading and has not been taken up since. Complex stability was followed under the transfection conditions by recording UV spectra at intervals; the spectra of the C2 and C8 variants did not change appreciably over time, whereas those of all of the other compounds lost intensity.^16^ This is the only time-resolved measurement in the DAPEG record. It is consistent with a rate-limiting step located after complex formation, and it places C2, the most active compound at the two higher charge ratios, in the same stability class as C8 rather than with its immediate neighbors in chain length. Loss of absorbance is not itself a measure of dissociation or of aggregation; therefore, the observation constrains the mechanism without identifying it.

The ordering that does emerge is stoichiometric, rather than structural. At the lowest charge ratio tested, 1.5:1, the most lipophilic variant, C16, gave the highest observed activity; at 3:1 and at 9:1, the least lipophilic, C2, gave the highest, with C6 and C10 reaching the leading group only at 9:1. C12 was not the highest at any ratio tested. One reading, offered as a hypothesis and not as a conclusion, is that when cationic charge is the limiting quantity, the contribution of the acyl chain to membrane interaction dominates, whereas once charge is in excess, the penalty attached to a long chain, tighter condensation, and correspondingly slower dissociation for cargo release becomes the binding constraint. No release or escape measurements were made; therefore, impaired endosomal dissociation remains an inference from the activity pattern alone. We propose, as a hypothesis rather than a conclusion, that the C2 variant represents a separate regime within group I in which the acyl group contributes little to membrane interaction and the chemistry behaves predominantly as a polycationic condenser, accounting for its activity across the charge ratios tested with the large plasmid DNA cargo but reduced relative performance expected with smaller cargoes such as siRNA.

This regime has a direct precedent in the closest historical analog of the DAPEG series, and the precedent also carries a warning. Futaki and colleagues report that stearyl-R_8_/plasmid complexes, formed at the 2:1 cation:anion ratio reported as optimal in that study, grow from approximately 1.4 μm at 3 min after mixing to above 5 μm at 30 min, the incubation time used before addition to cells, and nonetheless transfect COS-7 cells at Lipofectamine-comparable levels.^23^ Khalil and colleagues, measuring the same peptide and the same cargo but in serum-free DMEM at a forty-fold lower plasmid concentration, report 126 ± 2 nm.^24^ Micron-scale aggregation is therefore neither disqualifying for plasmid delivery nor an intrinsic property of a vector: it is a property of a vector under stated conditions.

A wider body of work on supramolecular peptide assemblies bears on this point. In sequence-defined peptide conjugates, small changes in residue volume and polarity propagate into the geometry of the assembled structure and hence into bulk material behavior,^51^ and coassembly of complementary peptides can send otherwise similar molecules to divergent nanostructural fates, broadening rather than narrowing the distribution of morphologies obtained.^52^ Oligo­(ethylene glycol) chains attached to a hydrophobic cargo likewise drive self-assembly into defined supramolecular polymers whose stability, and hence release behavior, is set by the linker chemistry rather than by the cargo.^53^ Read together, these results make the nonmonotonic chain-length dependence in the DAPEG series less anomalous: they describe systems in which molecular geometry and complementary interactions, not simply the strength of the driving interaction, determine higher-order structure. They also suggest that the DAPEG groupings would be better resolved by direct morphological methods than by scattering alone. The Group I and Group II assignments should likewise be read as condition-dependent, and the buffer, nucleic acid concentration, and time after mixing at which the DAPEG measurements were made should be stated explicitly whenever the two groups are invoked.

Two further reservations apply to the DLS data themselves. First, the 2 000–6 000 nm species are aggregates and should be described as such rather than as nanoparticles; micron-scale objects of this size are also liable to sediment on the time scale of the measurement and of the transfection experiment. Second, intensity-weighted DLS overweights the largest scatterers, so in a polydisperse population the reported z-average overstates the modal size; polydispersity indices are reported only in the supplementary Information and are not discussed alongside the size values in the main text, and the measurements were made in water rather than in the transfection medium, in which ionic strength and serum proteins would be expected to alter both size and colloidal stability. The reported DLS conditions are also difficult to reconcile with the transfection conditions: the compound concentration is given as 2.5 mg/mL, two to 3 orders of magnitude above the 0.61–3.64 μM used in the transfection experiments described in the same section. Either the two sets of conditions differ substantially, or the description is ambiguous; in either case the particle sizes should not be assumed to describe the species present during transfection. The two-group assignment should therefore be treated as a working classification derived from one technique under one set of conditions.

#### 3.5.3 Comparison with Peer Chemistries

Unlike polylysine, DAPEG presents a guanidinium headgroup, the same recognition motif as in oligoarginine CPPs, and the PEG arm interposes a flexible spacer between the cationic head and the backbone. This geometry differs from peptoids (where the side chain is appended directly to the backbone nitrogen without a spacer) and from PEGtides (where the PEG is the backbone and not the side arm).

#### 3.5.4 Testable Predictions

The model makes five predictions that can be tested with existing techniques and that, if borne out, would advance DAPEG from a proof-of-concept chemistry to a mechanistically understood vector. (i) Cryo-TEM imaging and polydispersity analysis across the C2–C16 series, at charge ratios matched to the transfection experiments, should corroborate the two-group assignment already indicated by DLS and establish how polydispersity varies across the series. (ii) Inhibitor-based uptake studies (chlorpromazine for clathrin-mediated endocytosis; filipin/methyl-β-cyclodextrin for caveolar/raft-mediated uptake; dynasore for dynamin-dependent pathways) should be applied to the DAPEG-DNA complex rather than to the free oligomer, since the free nonlipidated hexamers are already assigned to a clathrin- and actin-dependent route^14^ and the octaarginine record shows that a polyplex need not follow the portal used by its free peptide;^24^ the question is whether the complexes share that clathrin route, the lipid-raft and dynamin-dependent routes reported for lipitoids,^20^ or the macropinocytosis route that dominates for high-density octaarginine particles,^26^ and the sucrose/amiloride/filipin panel applied to the octaarginine particles provides a directly transferable protocol. (iii) Endosomal-escape assays using galectin recruitment reporters or pH-sensitive dyes should quantify the rate-limiting step of intracellular delivery; time-lapse imaging of pH-sensitive cargo labels has resolved endosomal pH change preceding escape and provides a usable readout.^10^ (iv) Time-resolved confocal microscopy with labeled DAPEG and labeled cargo should establish whether the vector cotraffics with the cargo to the cytoplasm or dissociates earlier. (v) Innate immune sensing (TLR3/TLR7/TLR9 reporters; type-I interferon induction) should be measured at therapeutically realistic doses, given that the chemistry has not yet been profiled for immunogenicity.

These predictions are explicit and are falsifiable. Confirmation of (i) and (ii), in particular, would convert the stoichiometry-dependent activity ranking from an empirical observation into a structure–activity rule with predictive value for the design of next-generation DAPEG analogs, including those carrying mRNA or ribonucleoprotein cargos.

### 3.6 Fate in Serum: What Peer Chemistries Predict and What Is Not Known

The PEG arms of DAPEG are an architectural element, not a stealth corona, and the distinction has consequences that the published record does not yet address. In PEGylated liposomes and in PEG-coronated polyplexes, the shielding function depends on a dense, high-molecular-weight PEG layer presented at the particle surface; corona molecular weight and architecture, rather than the presence of PEG as such, determine whether the effect is obtained. Jackson and colleagues compared six coronas grafted onto an identical pH-responsive polyplex core and found the effect to track corona molecular weight and architecture rather than corona chemistry. The 20 kDa Y-shaped PEG and the 20 kDa zwitterionic poly­(2-methacryloyloxyethyl phosphorylcholine) coronas were the least favorable for albumin adsorption by isothermal titration calorimetry, both significantly better than the 5 kDa linear PEG benchmark, and gave average circulation half-lives of 22 and 26 min against 5 to 8 min for every other polyplex in the library.^43^ The DAPEG side arm is a short heterobifunctional spacer carrying a guanidinium headgroup; it is neither of the length nor presented in the surface geometry that produces a stealth layer. Describing DAPEG as “PEGylated” in the pharmacological sense would therefore be a category error, and no serum-shielding function should be inferred from the acronym.

What, then, is the expected fate of a DAPEG-nucleic acid complex in serum? No direct evidence has been found. None of refs 12–16 assayed complex stability, protein adsorption, or transfection in the presence of serum, and the transfection experiments of Mallek and colleagues^16^ were performed without a serum challenge arm. The question can at present be addressed only by analogy to peer chemistries, and the analogy is not reassuring. Cationic nanocarrier-nucleic acid complexes that lack a shielding corona aggregate on contact with serum: Malcolm and colleagues reported that cationic nanoparticle-siRNA complexes aggregate at serum concentrations as low as 2%, with an approximately 4-fold reduction in cellular uptake, and that the pH-responsive membrane-lysis and complex-dissociation steps required for cargo release are themselves suppressed in serum.^44^ Comparable serum-induced aggregation is documented for polyethylenimine polyplexes.^45^ Because the lipidated DAPEG series carries a net positive zeta potential of +15 to +20 mV in its DNA complexes,^16^ it falls within the physicochemical class for which this behavior is reported. A serum challenge experiment across the C2–C16 series is therefore the least expensive informative addition to the DAPEG record, and it is logically prior to the in vivo study proposed in Section 5.

Whether a separate shielding element would be required for in vivo application is, on the present evidence, best answered as a design hypothesis with a known cost. PEGylation of cationic polyplexes does improve serum tolerance, but characteristically at the expense of cellular uptake: Santo and colleagues obtained a 350-fold higher transfection activity than branched PEI in the presence of serum with their best PEG-*b*-PAMA formulation, PEG45-*b*-PAMA168 at a nitrogen-counted N:P ratio of 25:1, while noting that PEG incorporation generally reduces uptake and hence transfection of target cells.^46^ Any shielding strategy for DAPEG would meet this trade-off directly, because the guanidinium arms that drive membrane interaction are the same elements that a corona would mask. A shielding element architecturally distinct from the guanidinium-bearing arms, a detachable corona, a zwitterionic block,^43^ or a ternary formulation is, on this reasoning, a more plausible direction than simply lengthening the existing arms or adding free PEG to the formulation. We emphasize that this is a hypothesis about design and not a result: no shielded DAPEG variant has been synthesized or tested.

A further consideration applies specifically to PEG-containing architectures and complicates any “add more PEG” answer. Anti-PEG antibodies are prevalent in the general population and drive the accelerated blood clearance phenomenon and complement activation-related pseudoallergy observed with PEGylated nanocarriers.^47^ Deuker and colleagues showed that anti-PEG antibodies become enriched in the protein corona of PEGylated nanocarriers and increase macrophage uptake, thereby counteracting the very stealth effect the PEG was introduced to provide.^48^ Whether the short PEG arms embedded in the DAPEG backbone present an epitope recognized by anti-PEG antibodies is an open question with direct bearing on the platform’s in vivo prospects, and it has not been tested. We name it here as a priority alongside the gaps listed in Section 5.1.

## 4 Comparative Summary

### 4.1 Methodological Heterogeneity and the Case for Standardized Reporting

The classes surveyed in Section 2 and the DAPEG record in Section 3 have not been generated under comparable experimental conditions. Three sources of heterogeneity dominate:

Cell lines and cargoes differ across the corpus. Peptoid-pDNA work has been benchmarked principally in standardized epithelial lines (HeLa, COS-7); lipitoid-siRNA work in primary fibroblasts; α/β-hybrid systems in HeLa with an anionic-lipid coating; PEGtides in airway (Calu-3) and neural (N41) lines; CARTs initially in HeLa and subsequently in primary T lymphocytes; stearylated CPPs in COS-7, NIH 3T3, and primary rat hippocampal neurons; PepFects in HeLa pLuc705 and mdx myotubes; lipidated DAPEG in HEK-293T 2D culture and spheroids. The cargoes range from SCOs (≈20 nt) and siRNA (≈21 bp duplex) through plasmid DNA (3–15 kb) to mRNA. Cross-platform inference is therefore constrained because the dominant rate-limiting step in delivery depends on cargo size and chemistry.

Reporting practice is not uniform. Particle size, zeta potential, cation-to-phosphate stoichiometry (reported as a charge ratio, an N:P ratio, or a molar or mass ratio MR, which are not interchangeable; Section 3.3), serum compatibility, optimal cargo dose, and quantitative cytotoxicity are reported inconsistently across the literature. Several otherwise informative entries in Tables 1 and 2 (Wagner peptide-PEG-Stp; PEGtide; α/β-hybrid) carry “not reported in cited source” entries for at least two of these parameters, not because the data are absent from the underlying experiments, but because the cited primary reports were not designed to enable cross-platform comparison. The frequency of these entries is itself the most concrete diagnostic of how far the field is from a comparable corpus.

Benchmarks are inconsistently chosen. Lipofectamine 2000 is the most frequently used positive control in the SCO and siRNA literature^27,30^ and remains the practical reference for assays of nucleic-acid delivery in HEK293, where approximately 91% eGFP-positive cells have been reported for eGFP-mRNA delivery at 2.5 μg mRNA, a figure that did not differ significantly across the 1, 2, 4, and 6 μL reagent volumes tested, with 2 μL sufficient for that cell line.^49^ Branched PEI, conventionally the 25 kDa material, is the more frequently used benchmark in the polymer literature; the reagent was introduced by Boussif and colleagues, whose dose–response series used the 800 kDa polymer.^17^ Neither of these two benchmarks has been used in the published DAPEG record (refs 12–16 ). The one commercial comparator that has been used is ViaFect, against which the nonlipidated hexamer matched fluorescence intensity at roughly half the cytotoxicity;^14^ the lipidated series has been compared only with untreated cells and with the nonlipidated parent. Table 2 collects the head-to-head data that are available in the cited literature and marks the entries for which direct DAPEG versus benchmark testing has not yet been done.

### 4.2 Cross-Platform Comparison

Table 1 summarizes the nine classes covered in Sections 2 and 3 along structural and operational dimensions. Table 2 collects quantitative metrics for the subset of systems whose cited sources report them. Entries marked *not reported in cited source* indicate that the parameter is either absent from the published record or has not been measured under conditions directly comparable to those of the other classes. The frequency of such entries is itself informative: quantitative cross-platform comparison in this field is hampered by methodological heterogeneity, and we identify this as one of the field-wide priorities in Section 5.

Table 2 collects the quantitative parameters reported in the cited primary literature for representative members of each class, together with two conventional benchmarks (Lipofectamine 2000, PEI 25 kDa) and the lipidated DAPEG series of Mallek and colleagues (2025; ref^16^). The table is restricted to entries for which the cited primary source provides at least three of the four core parameters (cell line, cargo, charge ratio or molar ratio, quantitative outcome). Where the published record reports only qualitative outcomes (“effective knockdown”, “outperformed commercial controls”), this is preserved as such; the entry is not converted to a numerical estimate. This is the most quantitative cross-platform comparison the literature presently supports, and the gaps it reveals are addressed in Section 4.1.

## 5 Outlook

Three priorities will determine whether peptidomimetic CPPs reach clinical translation. Methodologically, the field needs standardized mechanistic assays (pathway profiling, endosomal-escape quantification, immunogenicity panels) to enable cross-platform comparisons. The frequency of *“not reported in cited source”* entries in Tables 1 and 2 indicates how far the field is from such standardization. Translationally, in vivo evidence is still scarce for most platforms, including DAPEG. Chemically, integration of stimuli-responsive functionalities (for example, pH-responsive Dap chemistry, as developed by Tanishiki and colleagues for anticancer peptides)^50^ and convergence with targeting ligands offer the clearest paths forward.

For the DAPEG concept specifically, three concrete next experiments are within the laboratory’s reach and would close the most consequential gaps: (i) DLS and cryo-TEM characterization of the C2–C16 lipidated DAPEG-DNA complexes across the same charge ratio range to test the nonmonotonic particle-size hypothesis of Section 3.5; (ii) inhibitor-based uptake mapping in HEK-293T cells, the line used for the lipidated series (using the panel of chlorpromazine, filipin, and dynasore), to identify the dominant internalization pathway and to allow direct comparison with the lipitoid record;^20^ and (iii) a first in vivo biodistribution study in a tumor-bearing mouse model, using radiolabeled or fluorescently labeled DAPEG, to establish whether the in vitro promise translates to a tractable pharmacokinetic profile. These three experiments directly address gaps (i) and (ii) as numbered in Section 3.4; the gap (v) on independent replication is best resolved through head-to-head benchmarking against a peer chemistry, such as PepFect14,^27,30^ by an independent laboratory.

Two emerging frontiers deserve mention: mRNA and self-amplifying RNA (saRNA) delivery to tissues where lipid nanoparticles perform poorly and CRISPR-Cas ribonucleoprotein delivery, where the structural fragility of the cargo may favor sequence-defined peptidomimetic vectors over polydisperse polymers.

### 5.1 Limitations and Unmet Needs

The DAPEG record assembled in Sections 3.1–3.6 is incomplete on six dimensions that, taken together, define the work required before the chemistry can be considered a validated transfection platform rather than a proof of concept. We named them explicitly here.

#### 5.1.1 In Vivo Data Are Absent

The five primary DAPEG publications cited (refs 12–16 ) report exclusively on in vitro experiments in 2D- and 3D-cultured human cell lines. No biodistribution, pharmacokinetic, immunogenicity, or efficacy data are available for any animal model. By contrast, the comparator classes summarized in Table 1, peptoids/lipitoids (rabbit vocal-fold), PepFects (systemic gene delivery in mice; in vivo keratinocyte mRNA), and CARTs (mice, multiple routes), have crossed into mammalian in vivo evaluation; the stearoylated arginine-rich series is represented in refs 23–26 by in vitro data only. This is the largest single gap in the DAPEG record.

#### 5.1.2 Mechanism of Cellular Entry Is Mapped Only for the Free Oligomer

Romanowska and colleagues applied 4 °C, chlorpromazine, cytochalasin D, and methyl-β-cyclodextrin to the fluorescent nonlipidated hexamers and assigned their uptake to a clathrin- and actin-dependent endocytic route.^14^ No equivalent panel has been run on a DAPEG-nucleic acid or DAPEG-protein complex, or on any lipidated variant, and the octaarginine record shows that this distinction is not academic: free stearyl-R_8_ and its plasmid polyplex enter by different routes.^24^ The endosomal-escape assays (galectin reporters, pH-sensitive dyes) that would locate the rate-limiting step have not been reported for any DAPEG compound, nor has the release of the nucleic-acid cargo from the complex been investigated.

#### 5.1.3 Immunogenicity Has Not Been Profiled

Type I interferon induction, TLR3/TLR7/TLR9 reporter assays, and complement activation panels have not been reported for DAPEG or its lipidated variants. For comparator chemistries, this is now standard because the immunogenic profile is one of the principal differentiators of peptide-derived vectors from lipid nanoparticles in the clinical context.

#### 5.1.4 Behavior in Serum Is Uncharacterized

No published DAPEG experiment includes a serum challenge arm, and the short PEG arms embedded in the backbone do not constitute a stealth corona (Section 3.6). Because the lipidated DAPEG-DNA complexes remain net cationic, the peer literature predicts serum-induced aggregation and loss of uptake; a serum challenge series across C2–C16 should precede any in vivo study.

#### 5.1.5 Independent Replication Is Not Yet on the Record

All five DAPEG-defining publications (refs 12–16 ) originate from a single laboratory at the University of Gdańsk. Reproduction of the synthesis, characterization, and biological activity by an independent group has not been reported as of May 2026. Independent replication of the chain-length SAR (ref^16^) on at least one comparator cargo (siRNA or mRNA) and in at least one comparator cell line would be the single most consequential next step for establishing the chemistry as a platform.

#### 5.1.6 Cargo Scope Is Narrow

Of the five primary DAPEG-related publications, two report protease substrates (refs^12^ and^13^), one reports active-enzyme delivery (ref^15^, β-galactosidase via streptavidin scaffolding), and two report plasmid DNA transfection (refs^14^ and^16^). siRNA and mRNA, the two cargo classes most actively investigated in the contemporary CPP and CART literature, have not been tested. The mechanistic interpretation advanced in Section 3.5 predicts cargo-size-dependent behavior of the lipidated DAPEG series; testing that prediction on small cargoes (siRNA, ribonucleoprotein, mRNA) is identified there as the most informative next experiment. A direct head-to-head benchmarking against PepFect14 ^27,30^ on a common SCO or mRNA cargo would simultaneously address the independent-replication and cargo-scope gaps.

These six gaps are not specific to DAPEG; the peptidomimetic CPP field as a whole carries variants of all six. They are stated here at the DAPEG level because the present topical review treats DAPEG as its primary case study and because explicit naming of the gaps is the precondition for the experimental program proposed in the closing paragraphs of this section.

## 6 Conclusions

Peptidomimetic chemistry has produced a structured family of transfection vectors with tunable proteolytic stability, charge geometry, and shielding. DAPEG exemplifies how a modular building block, 2,3-Dap with pendant PEG arms, can be applied across functional categories from fluorogenic substrates to plasmid DNA transfection within a single decade. The principal gaps in the DAPEG record (in vivo data, mechanism of cellular entry and endosomal escape, behavior in serum, immunogenicity, cargo scope, and independent replication) define the most informative directions for the next phase of work and apply, in varying degrees, to the peptidomimetic CPP field as a whole. The mechanistic model proposed in Section 3.5 is a two-group description resting on direct DLS evidence from Mallek and colleagues:^16^ chains up to C10 form micron-scale aggregates with plasmid DNA, while C12 to C16 form compact 200–400 nm particles. It is established at a single charge ratio and has not yet been connected to transfection ranking. It is nonetheless explicit, falsifiable, and within reach of standard physicochemical and cell-biological techniques. Testing it on cargoes of different sizes (siRNA, mRNA, and ribonucleoprotein) would convert the stoichiometry-dependent chain-length ranking from a single-cargo empirical observation into a predictive structure–activity rule.

## Abbreviations

CPP: cell-penetrating peptide
DAPEG: 2,3-diaminopropionic acid-polyethylene glycol
Dap: 2,3-diaminopropionic acid
DLS: dynamic light scattering
GET: glycosaminoglycan-enhanced transduction
ivDde: 1-(4,4-dimethyl-2,6-dioxocyclohex-1-ylidene)-3-methylbutyl
LNP: lipid nanoparticle
MR: molar or mass ratio
Mtt: 4-methyltrityl
NSP4: neutrophil serine protease 4
PEG: polyethylene glycol
PEI: polyethylenimine
pDNA: plasmid DNA
RGD: arginine-glycine-aspartate
SCO: splice-correcting oligonucleotide
siRNA: small interfering RNA
SPPS: solid-phase peptide synthesis
Stp: succinoyl-tetraethylene pentamine
